# Longitudinal analysis of microbiome composition in Ghanaians living with HIV-1

**DOI:** 10.3389/fmicb.2024.1359402

**Published:** 2024-02-15

**Authors:** Lucky Ronald Runtuwene, Prince Kofi Parbie, Taketoshi Mizutani, Aya Ishizaka, Saori Matsuoka, Christopher Zaab-Yen Abana, Dennis Kushitor, Evelyn Yayra Bonney, Sampson Badu Ofori, Hiroshi Kiyono, Koichi Ishikawa, William Kwabena Ampofo, Tetsuro Matano

**Affiliations:** ^1^AIDS Research Center, National Institute of Infectious Diseases, Tokyo, Japan; ^2^Graduate School of Frontier Sciences, The University of Tokyo, Kashiwa, Japan; ^3^Department of Virology, Noguchi Memorial Institute for Medical Research, University of Ghana, Accra, Ghana; ^4^Department of Medicine, Washington University School of Medicine in St. Louis, St. Louis, MO, United States; ^5^The Institute of Medical Science, The University of Tokyo, Tokyo, Japan; ^6^Department of Internal Medicine, Eastern Regional Hospital Koforidua, Ghana Health Service, Koforidua, Ghana; ^7^Institute for Global Prominent Research, Graduate School of Medicine, Chiba University, Chiba, Japan; ^8^Department of Medicine, Chiba University-University of California San Diego Center for Mucosal Immunology, Allergy and Vaccines (cMAV), University of California San Diego, San Diego, CA, United States

**Keywords:** HIV, PLWH, Ghana, longitudinal analysis, gut microbiome dysbiois

## Abstract

Human immunodeficiency virus (HIV) 1 infection is known to cause gut microbiota dysbiosis. Among the causes is the direct infection of HIV-1 in gut-resident CD4^+^ T cells, causing a cascade of phenomena resulting in the instability of the gut mucosa. The effect of HIV infection on gut microbiome dysbiosis remains unresolved despite antiretroviral therapy. Here, we show the results of a longitudinal study of microbiome analysis of people living with HIV (PLWH). We contrasted the diversity and composition of the microbiome of patients with HIV at the first and second time points (baseline_case and six months later follow-up_case, respectively) with those of healthy individuals (baseline_control). We found that despite low diversity indices in the follow-up_case, the abundance of some genera was recovered but not completely, similar to baseline_control. Some genera were consistently in high abundance in PLWH. Furthermore, we found that the CD4^+^ T-cell count and soluble CD14 level were significantly related to high and low diversity indices, respectively. We also found that the abundance of some genera was highly correlated with clinical features, especially with antiretroviral duration. This includes genera known to be correlated with worse HIV-1 progression (*Achromobacter* and *Stenotrophomonas*) and a genus associated with gut protection (*Akkermansia*). The fact that a protector of the gut and genera linked to a worse progression of HIV-1 are both enriched may signify that despite the improvement of clinical features, the gut mucosa remains compromised.

## Background

Human immunodeficiency virus (HIV) is still a worldwide health problem, with 38.4 million living with HIV worldwide. Approximately 1.5 million people acquired HIV, and 650,000 people died from HIV-related causes in 2021 (HIV.gov, 2022). There are still no vaccines available to prevent the disease. Nevertheless, life expectancy and health outcomes in people living with HIV (PLWH) have improved with the availability of combination antiretroviral therapy (ART) that controls HIV replication. HIV incidence has declined 32% since 2010 (HIV.gov, 2022). However, a cure is still not attainable due to the ability of the virus to persist in long-lived cells. These cells cannot be targeted with current ART or HIV-specific immune responses.

Persistence may also be caused by the gut microbiome and its metabolites (Koay et al., 2018; Crakes and Jiang, 2019; Ishizaka et al., 2021). They have been shown to promote inflammation and immune activation in HIV-infected adults. This inflammation and immune activation are correlated with the disruption of gut mucosal integrity. HIV targets gut-associated lymphoid tissues as major sites of viral transmission, replication and seeding, and CD4^+^ T-cell depletion (Khan et al., 2017). In the gut, altered T-cell homeostasis, particularly of CD4^+^ Th17 cells, coincides with disruption of the intestinal barrier, where tightly opposed enterocytes lose their adhesion to adjoining cells (Dandekar et al., 2010). When the intestinal barrier is disrupted, microbial products travel from the lumen into systemic circulation, accumulating in the liver and brain tissue and activating the immune system (Estes et al., 2010).

Furthermore, the effect of HIV infection on the dysbiosis of the gut microbiome remains unresolved despite ART therapy (Shacklett and Anton, 2010). HIV replication is effectively suppressed by ART, but HIV in the reservoirs cannot be eradicated and CD4^+^ T cells cannot be fully restored in the gut or peripheral tissues. The restoration of CD4^+^ T cells in the gut is delayed compared to those in the peripheral blood (Guadalupe et al., 2003). Initiation of ART fails to reduce chronic immune activation and markers of microbial translocation, such as lipopolysaccharide (LPS) and soluble CD14 (sCD14) (Wallet et al., 2010). The interaction between the host and microbe at the gut mucosal interface is critical in preventing microbial translocation and immunity activation in HIV-infected individuals receiving suppressive ART.

Nevertheless, the elucidation of gut microbial interaction with immune activation is not an easy task, as gut microbiome composition is different all over the world (de Filippo et al., 2010; Brito et al., 2016; He et al., 2018). We previously added to the collective knowledge the results of the microbiome analysis in healthy Ghanaians (Parbie et al., 2021a) and compared it with that in Ghanaian PLWH (Parbie et al., 2021b). Through microbiome analysis on our cohort, we found that our results were mostly in line with previous reports, that is, a significant increase in the abundance of *Proteobacteria* but a decrease in the abundances of *Firmicutes* and *Bacteroidetes* at the phylum level, similar to previous observations in other countries (McHardy et al., 2013; Vujkovic-Cvijin et al., 2013; Monaco et al., 2016). However, some bacteria belonging to *Firmicutes*, namely, *Streptococcus*, *Dorea*, and *Blautia,* are enriched in PLWH (Parbie et al., 2021b). We note that this is different from several other reports, in which the abundance of *Lachnospiraceae*, including *Dorea* and *Blautia*, is decreased in HIV-1-infected individuals (Mutlu et al., 2014; Vázquez-Castellanos et al., 2015). Furthermore, we found a depletion of *Prevotella* in PLWH in Ghana, whereas previous studies on the gut microbiome have described enrichment of *Prevotella* in HIV-1-infected populations (Lozupone et al., 2013; Dillon et al., 2014).

To further complement the data, here, we describe the results of a longitudinal microbiome analysis of PLWH in Ghana.

## Materials and methods

### Study population

Data on HIV-1-negative (HIV-) and HIV-1-positive (HIV+) status at baseline (baseline_control and baseline_case, respectively) were described in Table 1. We enrolled matching pairs of HIV+ and HIV- individuals. Participants in the study were HIV+ individuals attending the Eastern Regional Hospital, Koforidua (RHK) in Ghana. A community health screening in these communities recruited HIV- individuals. We matched seronegative individuals by age (± 2 years), sex, and community of residence to serve as controls. Only adults above 18 years old were enrolled in this study. Seronegative participants who took antibiotics within four weeks of sample collection were not enrolled. Approximately 6 months after their baseline visit, stool samples were collected from HIV+ individuals, which represent the follow-up_case. CD4^+^ T-cell count and viral load were not measured on their second visit.

**Table 1:** Clinical and demographic characteristics.

Description	HIV-1-negative (*n* = 41)	HIV-1-positive (*n* = 47)	*p*-value^1^
Age (years): median (IQR^2^)	45 (19–73)	46 (18–75)	0.934
Gender: female number (%)	34 (82.93%)	37 (78.72%)	>0.99
CD4^+^ T-cell count (cells/μL): median (IQR^2^)	ND^3^	434 (31–1,384)	ND^3^
ART duration (months): median (IQR^2^) *n* = 37	ND^3^	54 (6–142)	ND^3^
Viral load (copies/mL): median (IQR^2^) *n* = 38	ND^3^	3,729 (20–200,936)	ND^3^
sCD14 (μg/mL): mean ± SD	0.503 ± 0.443	0.794 ± 0.791	0.238
IFABP (ng/mL): mean ± SD	0.987 ± 0.557	1.409 ± 0.898	0.042
LBP (μg/mL): mean ± SD	4.754 ± 2.14	5.096 ± 2.153	0.252

1 Statistical comparison between seronegative controls and HIV-1 infected individuals was performed by Wilcoxon rank sum test or Fisher’s exact test for gender.

2 Interquartile range.

3 Not determined.

RT, antiretroviral therapy; sCD14, soluble CD14; IFABP, intestinal free acid binding protein; LBP, lipoprotein binding protein.

### Sample collection

We collected venous blood and stool samples from enrolled participants. The total of 135 samples includes 41 samples from HIV- individuals (baseline_controls), and 94 samples from HIV+ individuals (n = 47) at baseline (baseline_case) and six months later at follow-up time point (follow-up_case). In the 24 h following their collection, all biological samples were transported to the Noguchi Memorial Institute for Medical Research (NMIMR) in Ghana, where they were processed for storage. We prepared plasma and peripheral blood mononuclear cells from venous blood. Stool samples were collected and stored at −80°C until DNA extraction.

### Bacterial fraction preparation from fecal samples

One gram of stool was washed with 3 mL of SM-plus buffer (100 mM NaCl, 50 mM Tris–HCl [pH 7.4], 8 mM MgSO4-7H2O, 5 mM CaCl2-2H2O, 0.01% [w/v] gelatin) and centrifuged at 6,000 × g for 5 min. This process was done for additional two times. Pellets were resuspended in 20 mL of SM-plus buffer and filtered through a 100-μm cell strainer (Corning, Tokyo, Japan). One milliliter of the filtered 20 mL of bacterial suspension was used for DNA extraction.

### DNA extraction, amplification, and 16S rRNA gene sequencing

DNA was extracted from the fecal sample-derived bacterial fraction as previously described (Kim et al., 2013). Gene libraries for the hypervariable V3-V4 region of 16S rRNA were prepared as previously described (Parbie et al., 2021b) according to the 16S Metagenomic Sequencing Library Preparation guide (Illumina, San Diego, United States; Part # 15044223 Rev. B). Sequencing was performed on the Illumina MiSeq using MiSeq Reagent Kit v3 (600-cycle) with a 20% PhiX (Illumina) spike-in at Noguchi Memorial Institute for Medical Research, Ghana. Sequencing was performed in five batches.

### Sequence analyses

Sequences were quality filtered, denoised, and analyzed with Quantitative Insights Into Microbial Ecology (QIIME 2 version 2022.2) (Bolyen et al., 2019). Briefly, raw paired-end reads were imported, demultiplexed, and quality filtered using the q2-demux plugin followed by denoising with DADA2 (Callahan et al., 2016) (via q2-dada2). Taxonomy was assigned to the resulting amplicon sequence variants against the SILVA database (release 138) (Quast et al., 2013) (via q2-feature-classifier). A phylogeny was constructed with the q2-phylogeny plugin. Batch effect was removed with an R package, MMUPHin (Ma et al., 2022) with the adjust_batch() function. Principal coordinate analysis calculated with Curtis-Bray, Unweighted UniFrac, and Weighted UniFrac distances, alpha diversity and microbial composition analyses were conducted with phyloseq (McMurdie and Holmes, 2013). Differentially abundant taxa by HIV-1 status were identified using the linear discriminant analysis (LDA) effect size (LEfSe) method (Segata et al., 2011) and the Analysis of Composition of Microbiome (ANCOM) (Mandal et al., 2015) approach with default parameters. Metagenomic pathway prediction was performed with Phylogenetic Investigation of Communities by Reconstruction of Unobserved States (PICRUSt2) (Douglas et al., 2020) with default parameters. We analyzed only results associated with the MetaCyc database (Caspi et al., 2020).

### CD4^+^ T-cell count and viral load measurement

CD4^+^ T-cell count was performed with BD FACSCount CD4^+^ reagents kit following manufacturer’s instructions. Viral load was measured with AmpliPrep/ COBAS TaqMan HIV-1 Test V2.0 following manufacturer’s instructions.

### Analysis of plasma markers for microbial translocation

An enzyme-linked immunosorbent assay (ELISA) was used to measure plasma lipopolysaccharide-binding protein (LBP), soluble CD14 (sCD14), and intestinal fatty acid-binding protein (I-FABP) levels (R&D Systems, Minneapolis, MN, United States).

### Statistical analyses

Data were obtained from 2017 to 2019. R 3.6.0 packages were used for statistical analyses. Comparison between categorical variables between groups was performed with the Wilcoxon using an R package, ggpubr with geom_pwc() function (Kassambara, 2023). *p* values less than 0.05 were considered significant. To test associations involving bacterial taxa showing significant differences in abundance by HIV-1 status and clinical and immunological markers, correlation analysis was performed using the R package Microbiome Multivariable Associations with Linear Models (MaAsLin2) (Mallick et al., 2021) using the following modifiers: analysis_method = “NEGBIN”, normalization = “NONE”, transform = “NONE”, max_significance = 0.5, min_prevalence = 0.5, min_abundance = 0.0001. PICRUSt2 data were visualized with Statistical Analysis of Taxonomic and Functional Profiles (STAMP) (Parks et al., 2014). Significant pathways between two groups (baseline_case vs. baseline_control and baseline_case vs. follow-up_case) were calculated with two-sided Welch’s t test and Bonferroni correction with a p value filter of 0.05.

## Results

### Microbial diversity of some genera rebound in PLWH at the second time point

**Figure 1:**
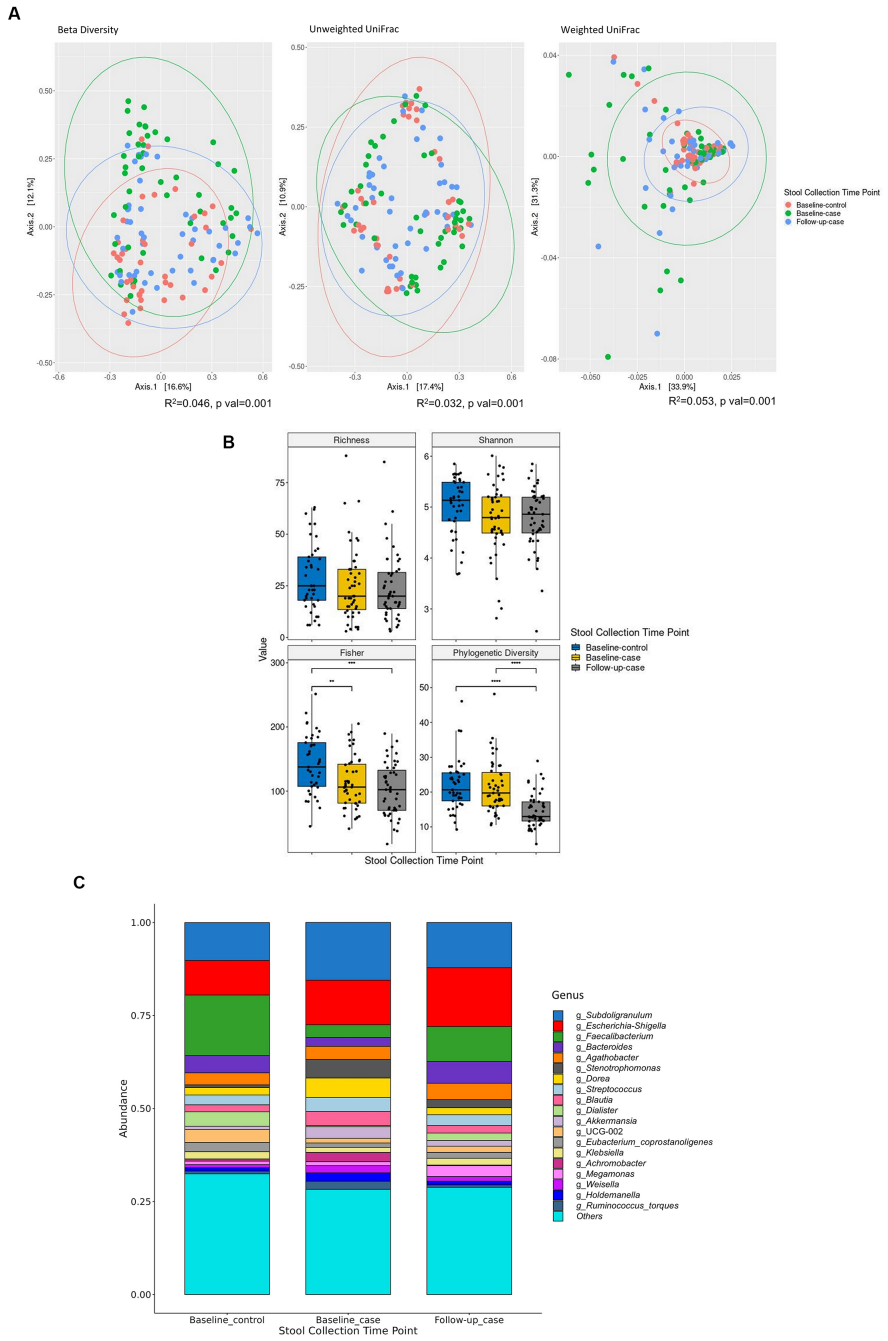
Alpha and beta diversity of the cohort. Principal component analysis shows that the microbial abundance of each of the three condition-timepoints can be clustered together. Each dot represents each sample’s taxon abundance in two dimensions. Beta diversity is measured using Curtis-Bray, Unweighted UniFrac, and Weighted UniFrac distances **(A)**. **(B)** Shows the diversity indices between condition-timepoint. The relative abundance of the 19 highest expressed genera in each condition-timepoint is shown in **(C)**. Statistical significance is calculated by the Wilcoxon test. **p < = 0.01, ***p < = 0.001, ****p < = 0.0001.

The total of 135 samples includes 41 samples from HIV- individuals (baseline_control), and 94 samples from HIV+ individuals (*n* = 47) at baseline (baseline_case) and six months later at follow-up time point (follow-up_case). Beta diversity shows that each condition-timepoint has a characteristic expression. Unweighted UniFrac shows that the species mostly overlap at all condition-timepoints. Weighted UniFrac, on the other hand, shows that the abundance of the species in follow-up_case is more similar to baseline_control than baseline_case (Figure 1A). We measured the alpha diversity of these condition-timepoints. Compared with baseline_control, all indices had lower diversity in baseline_case. Richness and Shannon were statistically insignificant, but two indices, i.e., Fisher and Faith’s phylogenetic diversity (PD) (Figure 1B). In the follow-up case, the microbial diversity indices were similar to those in the baseline case. Nevertheless, Faith’s PD shows a significant reduction at the second time point (Figure 1B). Compositional plot, which shows the microbial composition for each condition-timepoint, revealed that several genera were up-or downregulated in the follow-up_case compared to the baseline_case, with relative amounts comparable to the baseline_control (Figure 1C). For example, the genera *Subdoligranulum*, *Dorea*, and *Blautia* were less abundant in the follow-up case. On the other hand, the genus *Faecalibacterium* were more abundant in the follow-up case.

**Figure 2:**
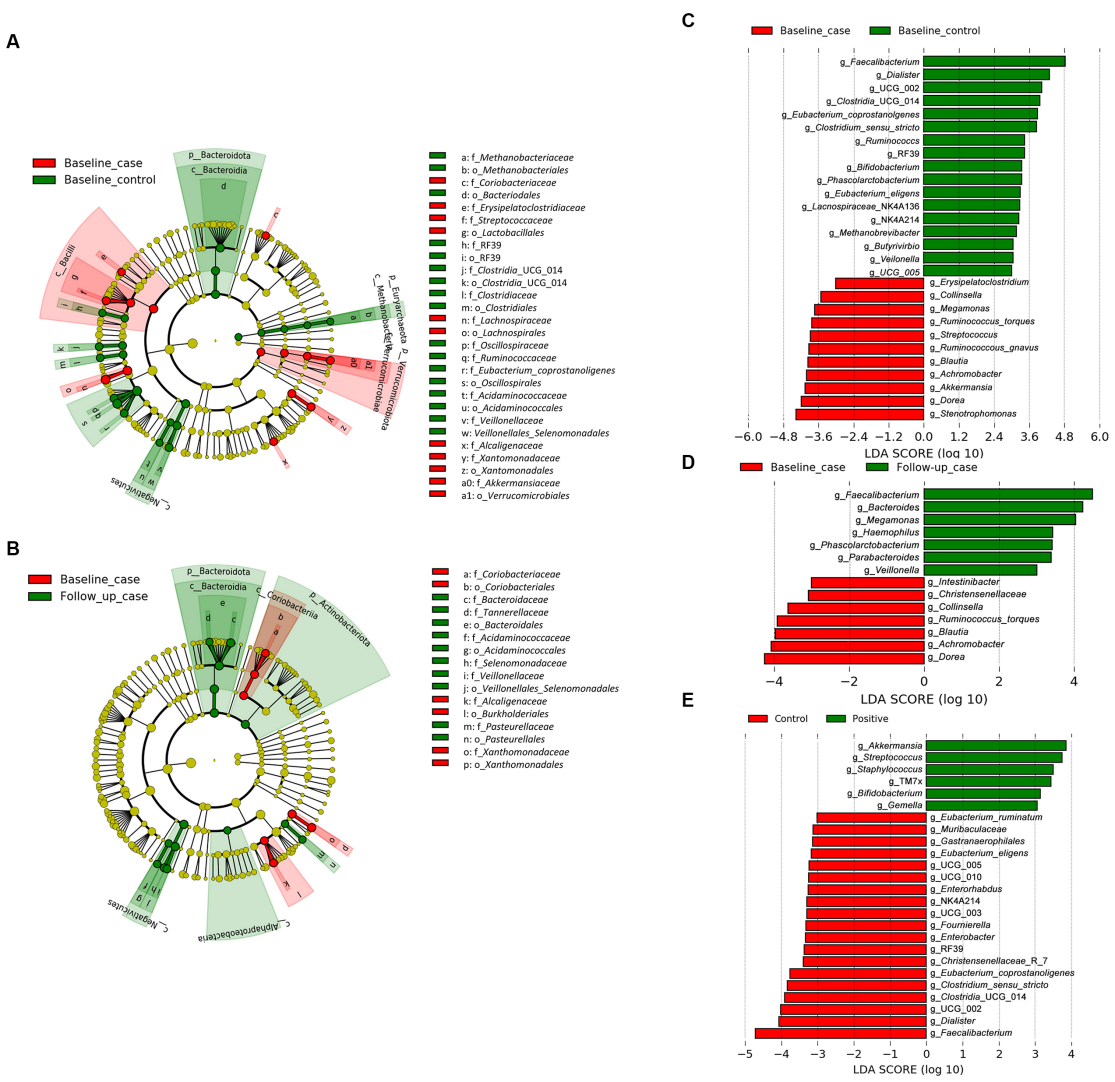
The result of linear discriminant analysis (LDA) effect size (LEfSe) analyses. LEfSe analysis between baseline_case and baseline_control and baseline_case and follow-up_case is shown in **(A,B)**, respectively. The difference in genera abundance between baseline_case and baseline_control and baseline_case and follow-up_case is shown in **(C,D)**, respectively. The difference in genera abundance between HIV negative and positive is shown in **(E)**. LDA threshold is 2.5.

We employed the linear discriminant analysis (LDA) effect size (LEfSe) method to identify the taxa characterizing each condition-timepoint (Segata et al., 2011). The phyla *Bacteriodota* and *Euryarchaeota* (archaea) were enriched in healthy individuals (baseline_control), while *Verucomicrobiota* was abundant in PLWH (baseline_case) (Figure 2A). Within the phylum *Firmicutes* (or *Bacillota*), there was abundant diversity. For example, the class *Negativicutes* was enriched in the baseline_control, while *Bacilli* was abundant in the baseline_case. Furthermore, there seems to be an intersection of abundance of baseline_control and follow-up_case, i.e., the phylum *Bacteriodota* and class *Negativicutes* (Figure 2B), which are abundant in both baseline_control and follow-up_case.

The genera *Dorea* and *Blautia* were increased in PLWH at baseline_case (Figure 2C), as well as *Achromobacter* and *Stenotrophomonas*, compared with baseline_control. Some genera in healthy people (baseline_control), such as *Faecalibacterium* and *Phascolarctobacterium* (Figure 2C) were also abundant in the follow-up_case (Figure 2D), corroborating the apparent reversion of the abundance of these genera. Nevertheless, some genera were highly abundant in PLWH regardless of time points, such as *Streptococcus*, *Staphylococcus*, and *Gemella* (Figure 2E). Analysis with ANCOM corroborates the LEfSe results, showing that the abundance in the baseline case and follow-up case was different: the genera *Achromobacter* and *Phascolarctobacterium* were abundant in the baseline case and follow-up case, respectively (Supplementary Figure 1).

**Figure 3:**
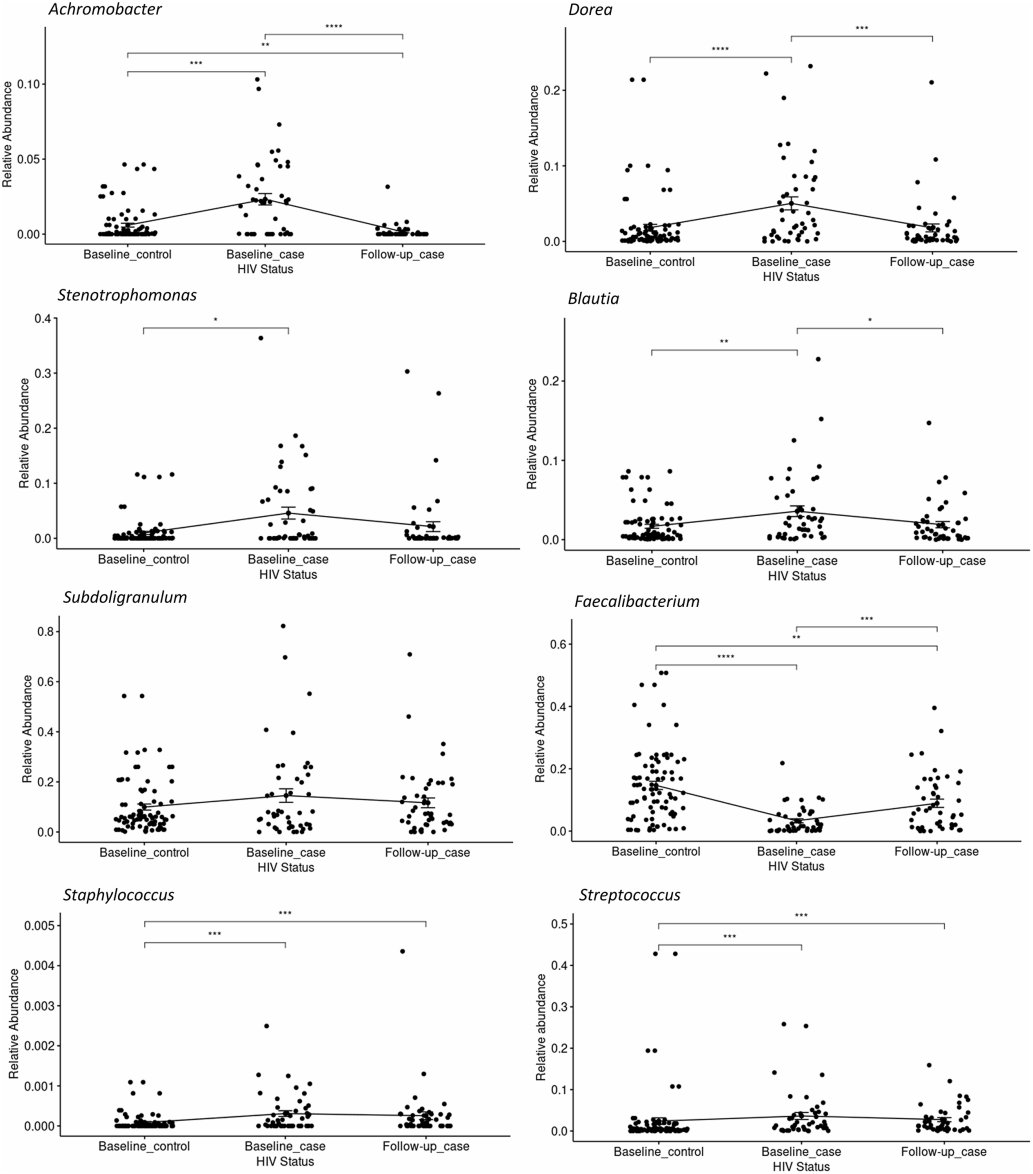
The relative abundance of select genera in each condition-timepoint. The genera *Achromobacter*, *Dorea*, *Stenotrophomonas*, *Blautia*, and *Subdoligranulum* shows low abundance in the baseline _control, high abundance in the baseline_case, and low abundance in the follow-up_case, albeit a non-significance in the genus *Subdoligranulum*. In contrast, the genus *Faecalibacterium* has a high abundance in the baseline_control, a reduction in the baseline_case, and a rebound in the follow-up_case. The genera *Staphylococcus* and *Streptococcus*, however, have high abundance in positive cases regardless of time point. Statistical significance is calculated by the Wilcoxon test. **p < = 0.01, ***p < = 0.001, ****p < = 0.0001.

The relative abundance of significantly differentially abundant genera of each condition-timepoint are presented in detail in Figure 3. A reversal in the abundance of the *Faecalibacterium* from baseline_case to follow-up_case and a decrease in the abundance of the *Dorea* in PLWH at baseline and follow-up_case was observed. Further, the persistence abundance of *Streptococcus* and *Staphylococcus* in HIV-positive despite the time point.

### CD4^+^ T-Cell count and soluble CD14 are significantly correlated with diversity

**Figure 4:**
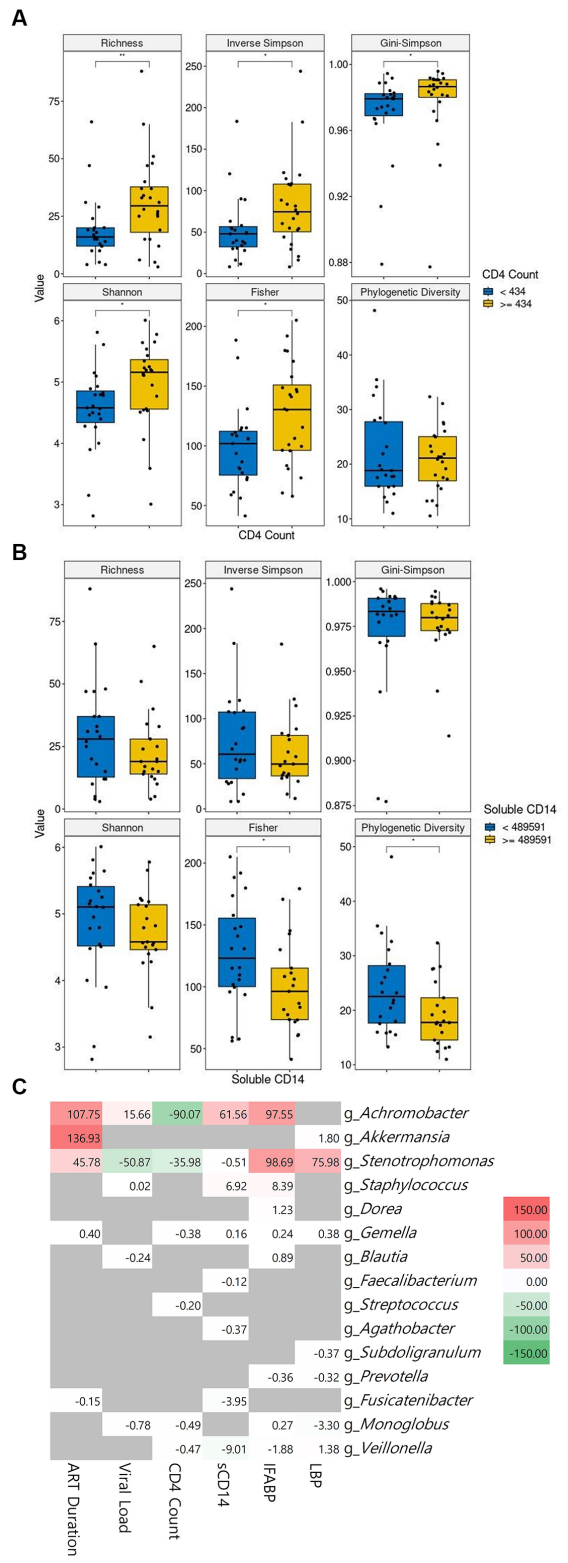
The correlation of microbiome abundance with clinical features. The diversity indices between low and high CD4^+^ cell count are shown in **(A)**. The diversity indices between low and high soluble CD14 level are shown in **(B)**. The cutoff value between high and low is the median value. CD4^+^ T-cell count is in cells/μL of blood and soluble CD4 is in pg/mL of blood. The correlation between genera and clinical features is observable in **(C)**. Statistical significance is calculated by the Wilcoxon test. **p < = 0.01, ***p < = 0.001, ****p < = 0.0001. ART: antiretroviral, sCD14: soluble CD14, IFABP: intestinal fatty acid binding protein, LBP: lipopolysaccharide-binding protein.

The correlation between clinical characteristics and alpha diversity at baseline continued to be examined. We conducted a comparative analysis of alpha diversity, splitting the PLWH population into two groups based on the median values of each of the six parameters, which were CD4^+^ T-cell count, viral load, ART duration, sCD14, IFABP, and LPS levels. The patients with high CD4^+^ T-cell counts showed significantly higher alpha diversity than the patients with low counts at baseline (Figure 4A). In contrast, a high sCD14 level showed significantly lower alpha diversity (Figure 4B). Longer ART duration was correlated with lower diversity, although it was not significant in our cohort (Supplementary Figure 2A). Higher IFABP levels were not significantly correlated with lower diversity (Supplementary Figure 2B). Viral load and LPS level did not seem to be correlated with diversity indices (Supplementary Figures 2C,D).

We then sought to link the abundance of microbial genera to the clinical features. Our cohort showed a positive correlation of *Achromobacter* with ART duration, viral load, sCD14, and IFABP and its inverse correlation with CD4^+^ T-cell count. Likewise, *Stenotrophomonas* followed the same pattern as *Achromobacter*, albeit with an inverse correlation with viral load. *Staphylococcus* showed a positive correlation with sCD14 and IFABP. Other genera did not show a meaningful correlation with the clinical features (Figure 4C). Interestingly, the correlation of ART duration with *Achromobacter* and *Sternotrophomonas* was positive. The abundance of the genus *Akkermansia* was also highly significantly related to this parameter.

### Metagenome pathway prediction software predicts a reduction of bacterial pathways activities at follow-up

**Figure 5:**
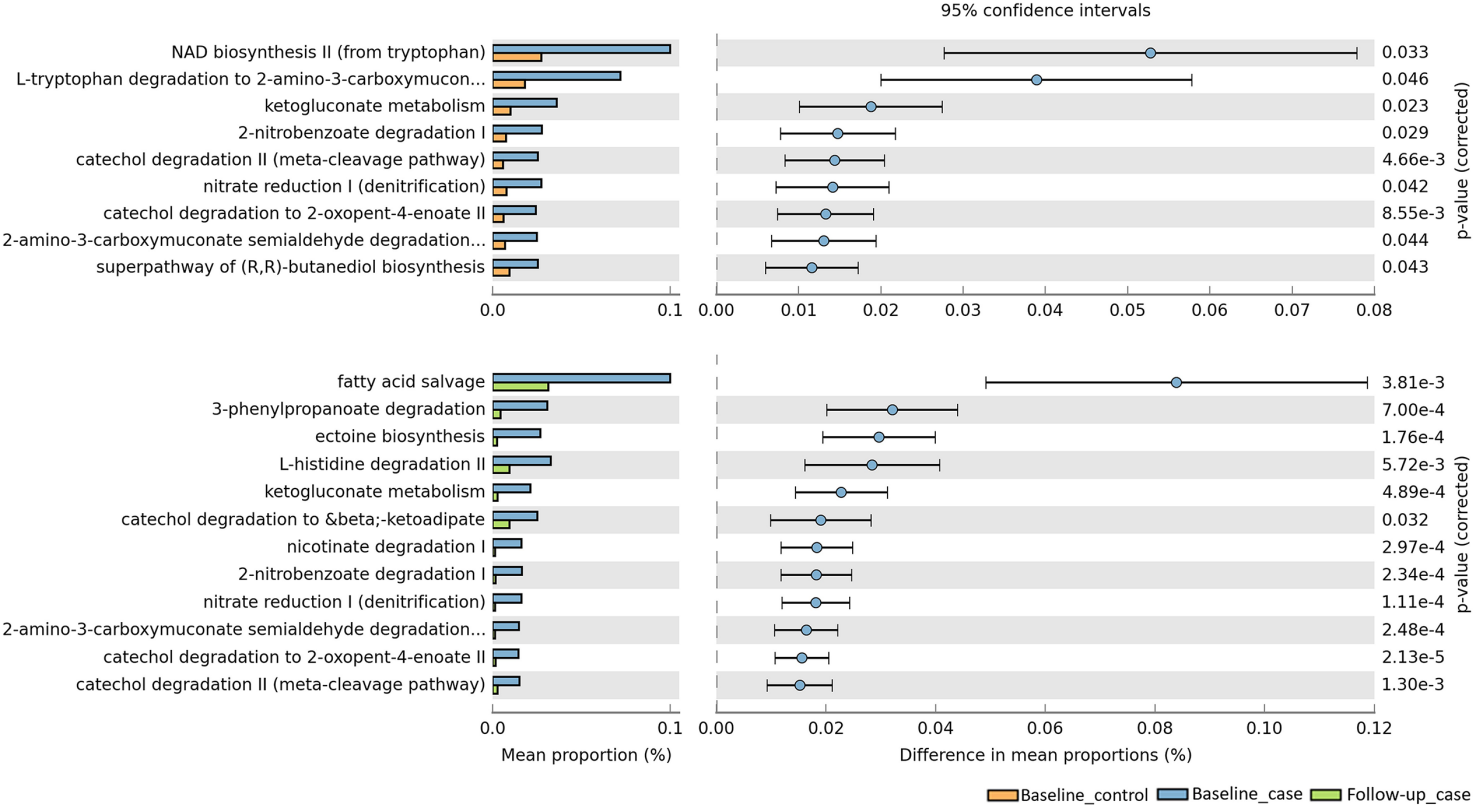
Predicted pathways upregulated/downregulated in our cohort. PICRUSt2 predicts that baseline_case samples have pathways that are significantly enriched compared to baseline_control and follow-up_case.

To evaluate the metabolic pathways, counting the functional genes directly following a shotgun metagenome sequencing is ideal. However, this needs a sequencer with a high sequencing capacity. In a situation where research limitations exist, we may use a prediction software. PICRUSt2 software was developed to predict the functional potential of a bacterial community based on marker gene sequencing profiles (Douglas et al., 2020). Utilizing this software, we compared the functional pathways based on the MetaCyc database (Caspi et al., 2020). Analysis revealed that bacterial pathways predicted a significant increase in the number of functional genes in baseline cases compared to baseline controls, but a decrease in the number of functional genes in follow-up cases compared to baseline cases (Figure 5). Exploring the MetaCyc database revealed the expected taxonomic range to possess the predicted pathways (Supplementary Table 1). While some of the pathways are not specific to bacteria, e.g., NAD biosynthesis II from tryptophan pathway is also found in eukaryotes, some pathways are found to be specific in bacteria, for instance ketogluconate degradation I, 2-nitrobenzoate degradation I, and fatty acid salvage pathways. Furthermore, some upregulated pathways are possessed by groups of bacteria known as pathogenic and opportunistic. Catechol degradation II, L-histidine degradation II, and nicotinate degradation I are pathways possessed by the phylum *Pseudomonadota*, whose members include the families *Neisseriaceae, Campylobacteriaceae, Enterobacteriaceae, Klebsiella, Escherichia, Burkholderiaceae, and Pseudomonadaceae* (Leão et al., 2023).

### Leaky gut during the treatment period

**Figure 6:**
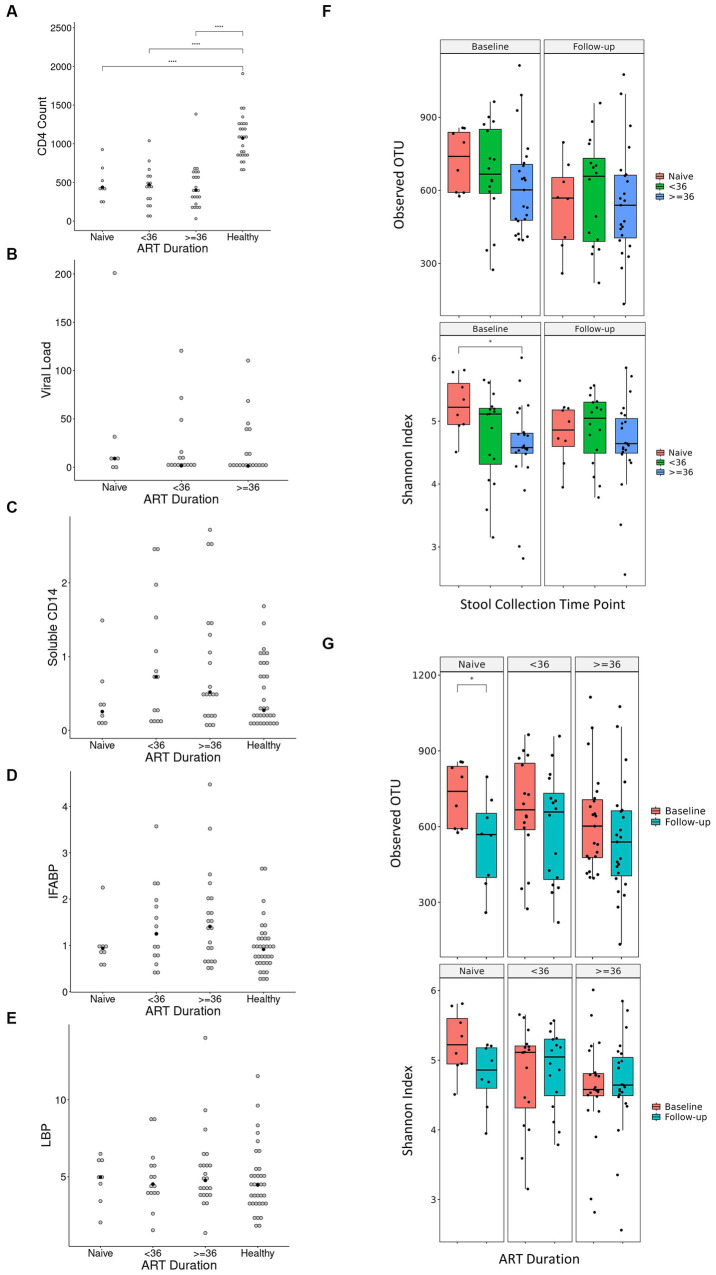
Diversity indices and clinical features in regard to treatment duration. Values of CD4^+^ T-cell count, viral load, soluble CD14, IFABP, and LBP in regard to treatment duration is shown in **(A–E)**, respectively. Antiretroviral (ART) duration is in months, CD4^+^ T-cell count is in cells/μL of blood, viral load is in copies/μL of blood, soluble CD14 is in μg/mL of blood, intestinal fatty acid binding protein (IFABP) is in ng/mL of blood, and lipopolysaccharide-binding protein (LBP) is in μg/mL of blood. Each white dot is the value of a sample while the black dots show the median value. Diversity indices in regard to duration treatment is shown in **(F,G)**. Statistical significance is calculated by the Wilcoxon test. **p < = 0.01, ***p < = 0.001, ****p < = 0.0001.

**Figure 7:**
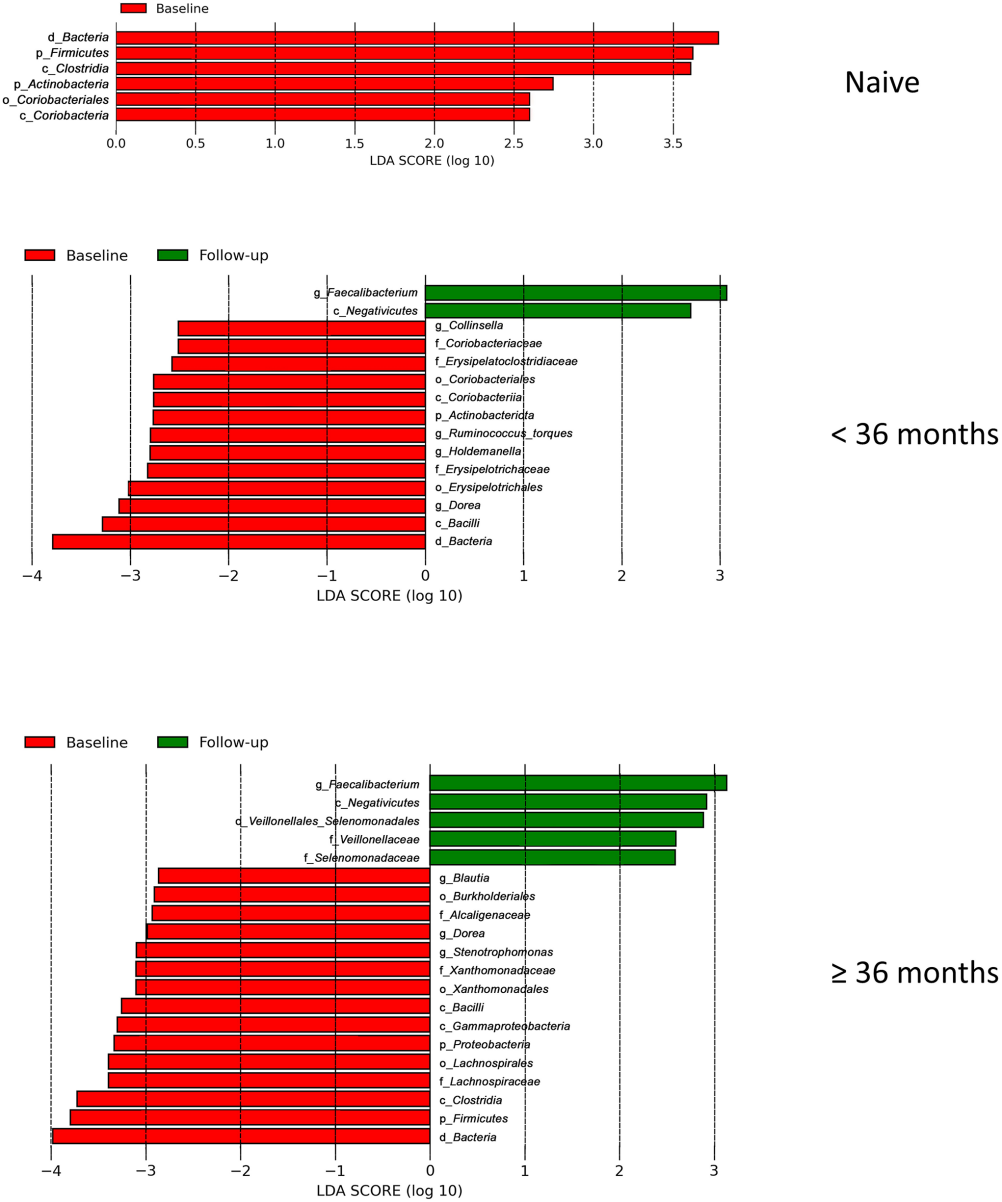
Linear discriminant analysis (LDA) effect size (LEfSe) in regard to treatment duration. LEfSe analysis between baseline_case and baseline_control and baseline_case and follow-up_case in regard to treatment duration is shown here. There is no follow-up result in naive due to its effect size is lower than the threshold 2.5.

Our finding that the abundance of both beneficial and pathogenic bacteria genera is highly correlated with the duration of ART motivated us to perform additional longitudinal analysis based on treatment duration. Here, we classified our cohort into ART-naïve, PLWH with ART treatment < 36 months, and PLWH with treatment ≥ 36 months. We found that the correlation between bacterial abundance and clinical features was stronger at treatment ≥36 months than at any other treatment duration (Supplementary Figure 3). CD4^+^ T-cell count showed that there was no change in cell number in all treatment duration (Figure 6A). Nevertheless, the median CD4^+^ cell counts of 434 cells/μL was well above 200 cells/μL for all treatment duration. Despite non-significance, viral load was in a decreasing trend with a median value of 3,729 copies/μL (Figure 6B). sCD14 and IFABP showed a higher median value than naïve at early treatment duration but was reduced at late therapy (sCD14, Figure 6C) or no change (LBP, Figure 6D). The median LBP value was of no change (Figure 6E). Nevertheless, sCD14, IFABP, and LBP values showed an increasing trend. Alpha diversity indices were lower in PLWH on treatment at baseline, with more profound significant reduction at late treatment period (Figure 6F). At follow-up, there was no index difference between the treatment conditions. Nevertheless, in comparison to baseline, diversity at follow-up was more significantly reduced in ART-naïve individuals (Figure 6G). LDA LEfSe showed abundance of genus *Faecalibacterium* and class *Negativicutes* on follow up at both <36 and ≥ 36 months ART treatment. The analysis could not identify a taxon with a strong effect size at follow-up in ART-naïve (Figure 7). Collectively these results revealed a leaky gut phenomenon at late treatment period despite relatively controlled disease progression with a mix of beneficial and pathogenic bacterial environment.

## Discussion

Our previous work confirmed that the gut microbiome composition of Ghanaian PLWH is mostly similar to other reports, with some discrepancies (Parbie et al., 2021a). *Faecalibacterium* as well as *Prevotella* and *Bacteroides* were not included in the top 10 abundant taxa in HIV+ compared to healthy individuals, while the genera *Achromobacter*, *Dorea*, *Blautia*, *Stenotrophomonas*, and *Streptococcus* were abundant, indicating loss of critical commensals in HIV-1-infected adults and proliferation of opportunistic pathogens (Parbie et al., 2021b). One discrepancy was that depletion of *Prevotella* in chronic HIV infection was not reported previously (Lozupone et al., 2013; Dillon et al., 2014; Mutlu et al., 2014; Vázquez-Castellanos et al., 2015). In addition, the abundance of *Dorea* and *Blautia* was not reported by the aforementioned authors. The discrepancies observed between this study and others may be explained by differences in inclusion and exclusion criteria, duration of ART treatment, sampling methods and sites, sample sizes, sequencing techniques, and statistical methods (Monaco et al., 2016). For example, the abundance of *Prevotella* is usually linked to MSM community, regardless of HIV status (Kelley et al., 2017; Armstrong et al., 2018; Vujkovic-Cvijin et al., 2020). This is in contrast to our cohort being mostly female.

Lower diversity indices in HIV positive samples have been reported before (Mutlu et al., 2014; Vázquez-Castellanos et al., 2015; Noguera-Julian et al., 2016), while some reports have disagreements (Dillon et al., 2014; Ling et al., 2016). We observed lower Shannon and Fisher indices in our PLWH cohort. These indices are related to low microbial diversity, which lead to the loss of gut integrity and increased microbial migration (Koay et al., 2018). The low microbial diversity was maintained at the second time point in this longitudinal analysis, with Faith’s PD index displaying an even lower value than the first time point.

Compositional plot, on the other hand, revealed a different story. At the second time point, the microbial composition showed a partial shift similar to that of healthy Ghanaians. This included abundance rebound of the phylum *Bacteroidota*, class *Negativicutes*, and genus *Faecalibacterium* as well as depletion of genera *Achromobacter*, *Dorea*, and *Blautia*. Some genera, such as *Streptococcus*, *Staphylococcus*, and *Gemella,* did not show this reversal and maintained their high abundance.

The genera *Faecalibacterium*, *Prevotella*, and *Bacteroides* are commensals commonly found in healthy individuals and are correlated with good health (Wexler, 2007; Lopez-Siles et al., 2017; Yeoh et al., 2022). On the other hand, the genera *Achromobacter*, *Streptococcus*, and *Stenotrophomonas* are opportunistic pathogens (Gordon et al., 2002; Calza et al., 2003; Tatro et al., 2014). *Dorea* and *Blautia* from the phylum *Firmicutes* and their correlation to good health are somewhat controversial (Vacca et al., 2020). We hypothesized that the reversal may be contributed by CD4^+^ T cells and sCD14, as these two clinical markers are correlated significantly with microbial diversity. Indeed, a lower CD4^+^ T-cell count in HIV+ patients is associated with gut dysbiosis, a reduced abundance of healthy *Ruminococcaceae* bacteria in the gut, and elevated serum proinflammatory cytokine levels (Guillén et al., 2019; Lu et al., 2021). sCD14, which is a marker of monocyte activation, is indirectly correlated with microbial translocation (Marchetti et al., 2013). Lower diversity in samples with high sCD14 levels may indicate the translocation of gut microbes to the bloodstream. In accordance to this, we found that a higher CD4^+^ T-cell count and lower sCD14 levels were correlated with higher diversity indices in this cohort.

Further, the genera *Achromobacter* and *Sternotrophomonas* were found to be highly correlated with the clinical features. We found that with the improvement of the markers, the abundance of these genera was significantly reduced. Indeed, lower CD4^+^ T-cell percentages, greater CD4^+^ T-cell activation, and higher sCD14 are correlated with the immune response to *Achromobacter xylosoxidans* (Tatro et al., 2014). The repletion of CD4^+^ regulatory T cells in gut-associated lymphoid tissue may help with the localization of these bacteria in Peyer’s patches and mesenteric lymph nodes. *Sternotrophomonas maltophilia* has been shown to induce T-cell exhaustion, which may further increase T-cell apoptosis (Wang et al., 2021). It is likely that the improvement of the immune system limits the vicious cycle caused by the bacteria through an unknown mechanism. We also found that the abundance of one genus, *Akkermansia*, was very significantly correlated with the antiretroviral duration. Considering the role of these bacteria in maintaining the health of the gut mucous (Ouyang et al., 2020), the abundance of *Akkermansia* is a sign that the bacteria are working to preserve the stability of the gut microbiome during disease progression. However, *Achromobacter* and *Stenotrophomonas* were also positively correlated with antiretroviral duration, corroborating the finding that gut mucous integrity is not consistently restored despite the improvement in plasma CD4^+^ T-cell count and reduction in anti-inflammatory cytokines (Li et al., 2016). One causing factor is CD4^+^ T-cell depletion in the terminal ileum, including Peyer’s patches, which is accompanied by irreversible fibrosis that disrupts normal gut mucosal function (Shacklett and Anton, 2010).

We also predicted the bacterial pathways likely to be enriched using prediction software. Comparison between baseline_control and follow-up_case to baseline_case reveals that samples from baseline_case likely have enrichment of pathways related to bacteria, some even exclusive to the phylum *Pseudomonadota.* The members of this phylum are known to be pathogens of the respiratory tract (*Achromobacter, Klebsiella, Pseudomonas, Sternotrophomonas*), gastrointestinal tract (*Aeromonas, Campylobacter, Escherichia, Helicobacter*), and blood (*Brevundimonas, Neisseria, Serratia, Stenotrophomonas*), among other conditions (*Citrobacter, Enterobacter, Haemophilus, Proteus*) (Leão et al., 2023). This implies that the activity of pathogenic bacteria was high in the baseline case. The activity seemed to be reduced to be similar to control subjects in the follow-up_case, although not completely.

Classifying our cohort into ART-naïve, PLWH with ART treatment <36 months, and ART treatment ≥36 months gives a deeper insight about our cohort. First, we were able to observe that the Ghanaian PLWH might have a relatively good response to therapy. Despite non-significance, CD4^+^ T-cell count seemed on the increasing trend with a median value above WHO cutoff of 200 cells/μL. Viral load was also on the decreasing trend with a median of 3,729 copies/μL, despite non-significance. Microbial translocation markers were on the increasing trend. This suggests that despite a somewhat controlled viremia and disease progression, leaky gut was occurring. This was corroborated with lower diversity in late treatment period and on follow-up of ART-naïve subjects. LDA LEfSe showed abundance of the genus *Faecalibacterium* and class *Negativicutes* on follow-up at both <36 and ≥ 36 months ART treatment, showing a mixed bacterial environment. *Negativicutes* is a Gram-negative class belonging to the phylum Firmicutes that is enriched in the gut microbiota of HIV-infected subjects with CD4^+^ T cell recovery higher than 500 cell/μL. Its abundance was positively correlated with several inflammation markers that mediate an LPS-induced immune response (Baltazar-Díaz et al., 2023).

Taken together, all of these results show that at the second time point, the gut microbial activity partially recovered but not completely, similar to that of healthy subjects. This includes the reduction and the enrichment of some genera correlated to worse and better HIV progression, respectively. This may suggest that the regiment therapy is somewhat effective in our cohort, although not optimal. Additionally, the significant correlation of *Akkermansia*, *Achromobacter*, and *Stenotrophomonas* with antiretroviral duration implies that gut integrity may still be compromised despite the improvement in CD4^+^ T-cell count and sCD14 during the treatment period. However, a longer ART duration does not necessarily mean a better gut microbial environment as we also found an increasing trend of microbial translocation markers at the late treatment period which resulting in a mixed bacterial environment in the gut.

The limitation of this research is that we could only analyze the correlation of clinical features with bacterial composition at one time point (baseline_case) because we could not collect clinical information, such as CD4^+^ T-cell count and viral load from the follow-up_case samples due to logistical problems. The reason was because we could not import the required reagents for the analysis in time. Furthermore, although the viral load range is very large, but the median viral load of 3,729 copies/mL may suggest that this data may only be applicable to PLWH with relatively low viral burden. Our data are also limited to perform additional analyses of bacterial composition in high and low values of the clinical features. Another concern is we could not rule out the time of infection to further classify the PLWH as recently or chronically infected. This limits the understanding that the time to treatment or the antiretroviral itself may have a direct effect in modifying the gut microbiome. At least one study showed that nucleoside reverse-transcriptase inhibitor (NRTI)-based antiretroviral therapy has a more suppressive impact on microbiota composition and diversity in the gut (Imahashi et al., 2021). Lastly, in the publications prior to this analysis, we found a strong correlation of ART treatment with antibiotic usage (Parbie et al., 2021b). As antibiotics have a direct effect in modifying gut microflora, we could not rule out the possibility of antibiotics reduced the diversity at late treatment period observed here.

In conclusion, we have presented here a longitudinal analysis of the gut microbiome composition in Ghanaian PLWH six months apart. We have shown that at the second time point, gut microbial activity was partially recovered, similar to that of healthy subjects, but not completely. Despite the relatively controlled disease, we found that genera correlated with both worse and better gut microbiomes were enriched and highly correlated with the duration of treatment, likely suggesting a volatile gut environment. This may support the theory that the gut microbiome does not recover despite controlled disease.

## Data availability statement

The datasets presented in this study can be found in online repositories. The names of the repository/repositories and accession number(s) can be found at: https://www.ddbj.nig.ac.jp/, DRA010770 https://www.ddbj.nig.ac.jp/, DRA017623.

## Ethics statement

The studies involving humans were approved by the Institutional Review Board of Noguchi Memorial Institute for Medical Research (NMIMR) and the Ethical Committee of National Institute of Infectious Diseases (NIID). The studies were conducted in accordance with the local legislation and institutional requirements. The participants provided their written informed consent to participate in this study.

## Author contributions

LR: Data curation, Formal analysis, Investigation, Methodology, Visualization, Writing – original draft, Writing – review & editing. PP: Data curation, Investigation, Methodology, Writing – review & editing. TaM: Data curation, Investigation, Methodology, Supervision, Writing – review & editing. AI: Writing – review & editing. SM: Formal analysis, Writing – review & editing. CA: Data curation, Writing – review & editing. DK: Data curation, Writing – review & editing. EB: Data curation, Writing – review & editing. SO: Data curation, Writing – review & editing. HK: Writing – review & editing. KI: Data curation, Methodology, Supervision, Writing – review & editing. WA: Funding acquisition, Supervision, Writing – review & editing. TeM: Funding acquisition, Supervision, Writing – review & editing.
