# Supplementary figures and images for: Longitudinal analysis of microbiome composition in Ghanaians living with HIV-1
Source: Front Microbiol. 2024 Feb 15;15:1359402. doi: 10.3389/fmicb.2024.1359402 (PMC10902004; doi:10.3389/fmicb.2024.1359402)

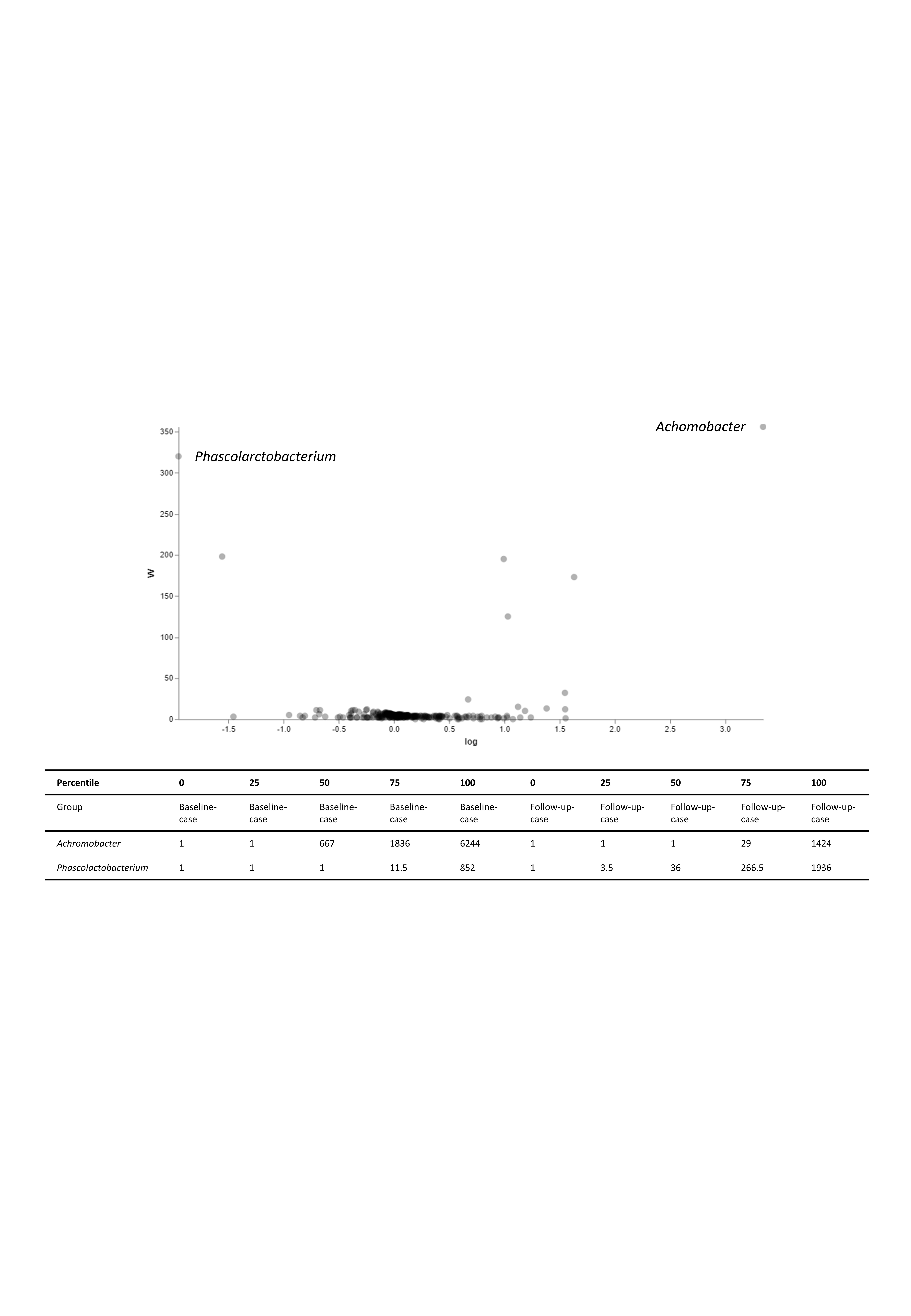

Supplement: Supplementary file 1 [file Image_1.JPEG]

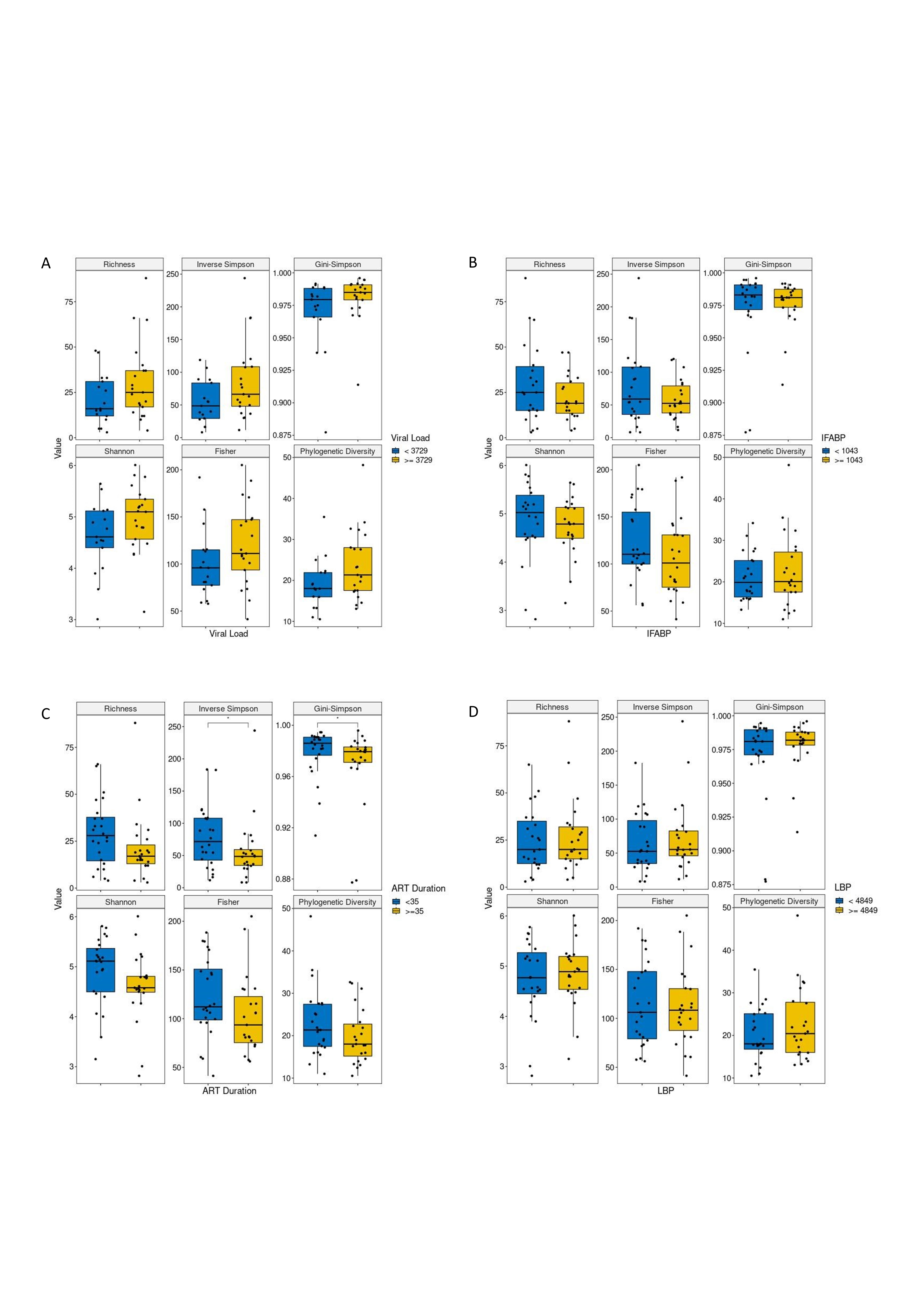

Supplement: Supplementary file 2 [file Image_2.JPEG]

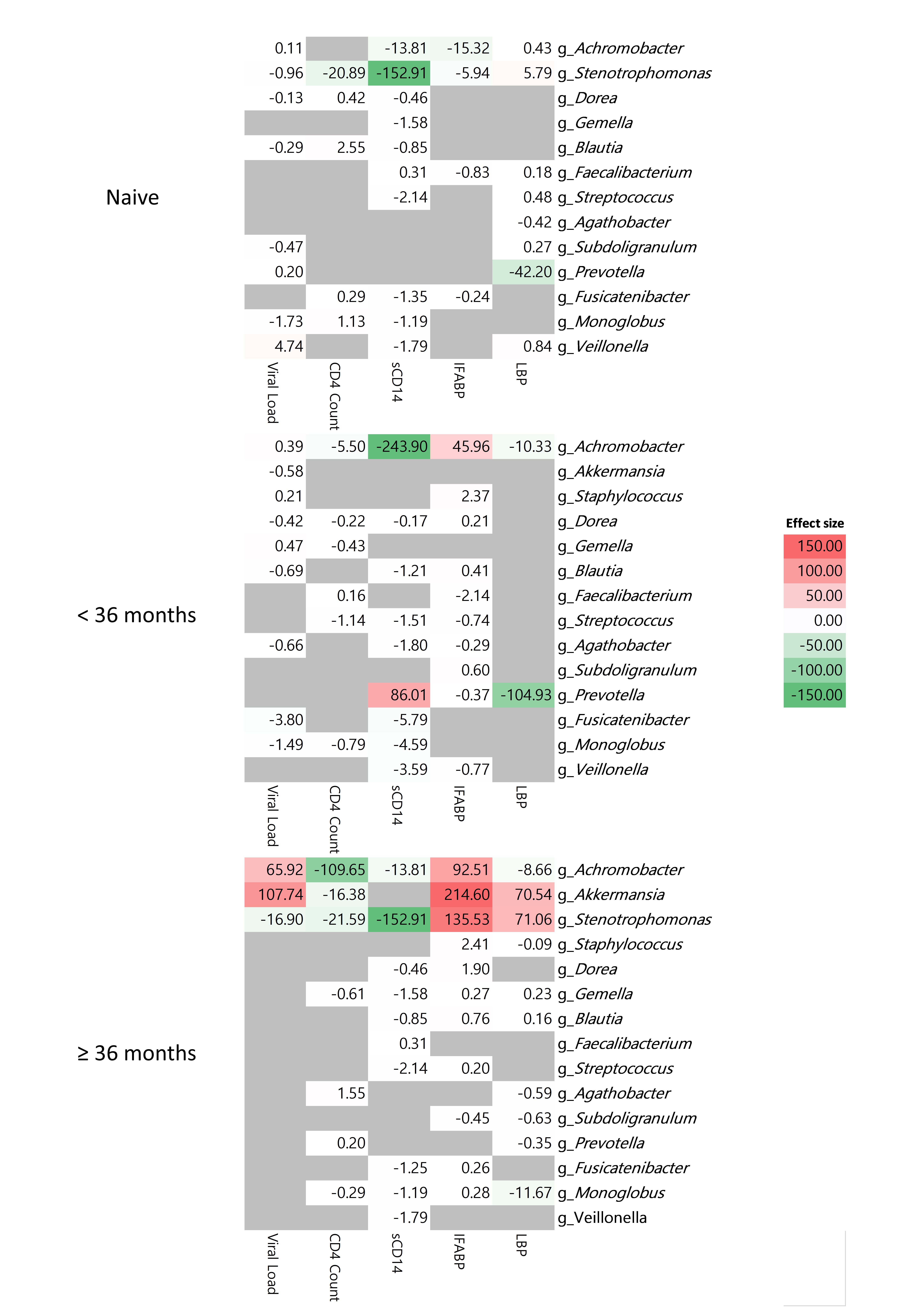

Supplement: Supplementary file 3 [file Image_3.JPEG]
