# Supplementary material for: Longitudinal analysis of microbiome composition in Ghanaians living with HIV-1
Source: Front Microbiol. 2024 Feb 15;15:1359402. doi: 10.3389/fmicb.2024.1359402 (PMC10902004; doi:10.3389/fmicb.2024.1359402)
Supplement: Supplementary file 4 [file Table_1.DOCX]

| Pathway  (Link to MetaCyc database) | Expected taxonomic range |
| --- | --- |
| NAD biosynthesis II (from tryptophan)  (https://metacyc.org/META/NEW-IMAGE?object=NADSYN-PWY&redirect=T) | *Bacteria*, *Eukaryota* |
| L-tryptophan degradation to 2-amino-3-carboxymuconate semialdehyde  (https://metacyc.org/META/NEW-IMAGE?object=PWY-5651&redirect=T) | *Bacteria*, *Fungi*, *Metazoa* |
| **Ketogluconate degradation I**  (http://metacyc.org/META/NEW-IMAGE?type=PATHWAY&object=KETOGLUCONMET-PWY&detail-level=2) | *Bacteria* |
| **2-nitrobenzoate degradation I**  (https://metacyc.org/META/NEW-IMAGE?type=PATHWAY&object=PWY-5647) | *Bacteria* |
| **Catechol degradation II (meta-cleavage pathway)**  (https://metacyc.org/META/NEW-IMAGE?object=PWY-5420&redirect=T) | *Actinomycetota*, *Pseudomonadota* |
| **Nitrate reduction I (denitrification)**  (https://metacyc.org/META/NEW-IMAGE?type=PATHWAY&object=DENITRIFICATION-PWY) | *Archaea*, *Bacteria*, *Fungi* |
| **Catechol degradation to 2-oxopent-4-enoate II**  (http://metacyc.org/META/NEW-IMAGE?type=NIL&object=PWY-5419&redirect=T) | *Actinomycetota*, *Pseudomonadota* |
| **2-amino-3-carboxymuconate semialdehyde degradation to 2-hydroxypentadienoate**  (http://metacyc.org/META/NEW-IMAGE?type=PATHWAY&object=PWY-5654) | *Bacteria* |
| Superpathway of (R,R)-butanediol biosynthesis  (http://metacyc.org/META/NEW-IMAGE?type=NIL&object=P125-PWY&redirect=T) | *Bacteria* |
| Fatty acid salvage  (http://metacyc.org/META/NEW-IMAGE?type=PATHWAY&object=PWY-7094) | *Bacteria* |
| 3-phenylpropanoate degradation  (http://metacyc.org/META/NEW-IMAGE?type=PATHWAY&object=P281-PWY) | *Archaea*, *Bacteria* |
| Ectoine biosynthesis  (http://metacyc.org/META/NEW-IMAGE?type=PATHWAY&object=P101-PWY) | *Bacteria* |
| L-histidine degradation II  (http://metacyc.org/META/NEW-IMAGE?type=PATHWAY&object=PWY-5028) | *Pseudomonadota* |
| Catechol degradation to beta-ketoadipate  (http://metacyc.org/META/NEW-IMAGE?type=PATHWAY&object=CATECHOL-ORTHO-CLEAVAGE-PWY) | *Actinomycetota*, *Pseudomonadota* |
| Nicotinate degradation I  (http://metacyc.org/META/NEW-IMAGE?type=PATHWAY&object=PWY-722&orgids=LEISH) | *Pseudomonadota* |

Supplementary Table 1. List of taxa known to possess the pathways predicted to be upregulated in baseline-case by PICRUSt2. Pathways marked with bold fonts are upregulated in baseline-case in comparison with both baseline-control and follow-up-case.
